# Mathematical Modeling of Acidity Removal from Natural Gas through Alkaline Scrubbing

**DOI:** 10.1021/acsomega.5c07091

**Published:** 2025-08-25

**Authors:** Raimundo A. L. Sobrinho, Walisson de J. Souza, Domingos F. de S. Souza, Antonio M. de O. Júnior, José J. Marques

**Affiliations:** † 74361 Santa Cruz State University, Ilhéus, BA 45662-900, Brazil; ‡ Chemical Engineering Department, 28125 Federal University of Rio de Janeiro, Rio de Janeiro, RJ 21941-853, Brazil; § 74391 Federal University of Sergipe, São Cristóvão 49107-230, Sergipe, Brazil; ∥ 28123 Federal University of Rio Grande do Norte, Natal 59078-970, Rio Grande do Norte, Brazil; ⊥ Tiradentes University, Institute of Technology and Research (ITP), Av. Murilo Dantas, 300, Aracaju, SE CEP 49032-490, Brazil

## Abstract

The production of natural gas (NG) and its contribution to the world energy matrix have increased in recent years. The use of natural gas, mainly stranded gas, usually requires pretreatment. This paper presents a mathematical model for reactive absorption in a bubble column containing a sodium hydroxide solution to remove sour components from natural gas. Model validation was based on experimental data by solving the system of equations via the computer package EMSO-Environment for Modeling, Simulation and Optimization. The results of the evaluation for some operating situations demonstrate that the model can be considered a suitable tool for accurately simulating the removal of H_2_S even in the presence of CO_2_ and provides a means to control the process on the basis of easily measured process variables, which are appropriate for the desulfurization of natural gas from stranded gas reserves.

## Graphical abstract

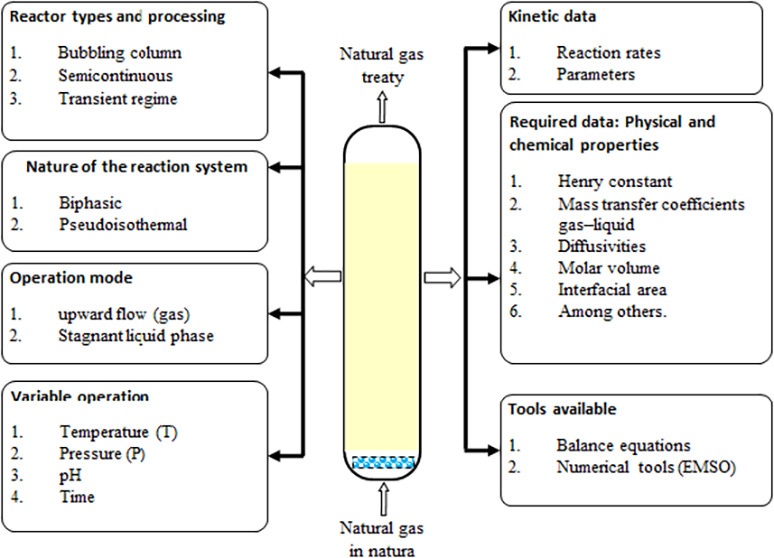

## 1 Introduction

Natural gas is a mixture of hydrocarbons trapped underground in a porous, permeable rock formation, associated with or not associated with oil, which remains in a gaseous state under normal atmospheric conditions and is produced directly from petroleum or gas reservoirs. It is composed primarily of methane, ethane and propane in small proportions, in addition to inorganic gases (e.g., hydrogen sulfide, carbon dioxide, nitrogen, and water).^1^ Sulfur compounds are undesirable because they increase the polarity of oil (increasing the stability of emulsions) and the corrosive properties of petroleum products, poisoning catalysts used in some industrial processes and determining the color and smell of the end products. They are toxic and can generate atmospheric pollutants such as SO_2_ and SO_3_, which form H_2_SO_3_ and H_2_SO_4_ (sulfuric acid) in aqueous media.^2^

Absorption in gas treatment processes involves the transfer of a substance from the gaseous phase to the liquid phase through the phase boundary.^3^ The absorbed material can be physically dissolved in the liquid or react chemically with the solvent. The absorption of acid gases is based on a standard operation, whose equipment involves variations of some type of tower, column or mixing vessel, such as a packed bed column, a spray tower, a bubbling column or a centrifugal contactor, in which the gaseous stream, which is usually in counterflow, is put into contact with the absorbing solution.^4^

Many studies have reported several methods for removing hydrogen sulfide and other acidic compounds from natural gas and biogas, most of which are based on reactive absorption in the aqueous phase. The main substances used as absorbents are sodium hydroxide and sodium carbonate.;^5,6^ alkanolamines and iron salts (III), combined or not with EDTA;^7–10^ and sodium phosphomolybdate,^11^ iron sulfate II (*FeSO*_4_), zinc sulfate (*ZnSO*_4_) and copper sulfate (*CuSO*_4_).^12^

There are few studies in the literature that focus on the mathematical modeling of natural gas desulfurization systems with inorganic absorbents, such as those reported by Bontozoglou and Karabelas,^13^ Meng et al.^14^ and Peng and Cao.^15^ However, most of these methods rely on mathematical modeling validation against experimental data, resulting in a lack of systematization in the study by assessing the isolated and combined effects of the variables involved in reactor performance. They are generally steady-state models that fail to represent the transient behavior that occurs in real operating situations. Thus, the present work consists of a theoretical and computational study on a dynamic kinetics model for the numerical investigation of natural gas alkaline washing using an aqueous sodium hydroxide solution by taking pH as the only parameter necessary to control the process and providing a means for developing an embedded technology for use in stranded areas, which is the main advantage of the process.

Although hydrogen sulfide (H_2_S) removal by alkaline absorption is a well-established process, there is a growing demand for kinetic models that enable dynamic control, field-level deployment, and simplified monitoring in real-time. Traditional models have focused on steady-state or equilibrium assumptions, which limit their predictive utility under fluctuating process conditions. Hashemi et al.^16^ used CFD modeling to simulate H_2_S and CO_2_ absorption in monoethanolamine (MEA), highlighting the influence of transport and thermal effects. Bontozoglou and Karabelas^13^ applied two-film theory to evaluate absorption in aqueous hydroxide and demonstrated the selective enhancement of H_2_S uptake due to slower CO_2_ kinetics.

Recent research extends beyond traditional acid–base chemistry to include oxidative and radical-based mechanisms. Ali et al.^17^ investigated thiolated arsenic oxidation under varying pH and metal ion concentrations-analogous to sulfide oxidation systems. Lai et al.^18^ characterized sulfate radical interactions with sulfur-containing organics, and Cao et al.^19^ examined disulfide-forming kinetics under diffusion-limited regimes. These studies collectively emphasize the need for models that account for reaction kinetics, mass transfer constraints, and operational control variables-gaps that our model directly addresses through a transient framework with pH as the core control parameter.

The proposed model was validated with experimental data by solving a system of equations aided by the computational package EMSO (*Environment for Modeling Simulation and Optimization*). Comparisons were made between the calculated and experimental results for the instantaneous reactive absorption of H_2_S in an aqueous solution of NaOH under different operating conditions. The model fit the experimental results appropriately, achieving the objective of the modeling.

### 1.1 State-of-the-Art of Natural Gas Desulphurization

The rigorous formulation of a mathematical model to represent the phenomena that occur in a biphasic reactor operating in turbulent flow under thermal effects is a difficult task because its intrinsic complexity once its behavior depends on the fluid characteristics, mass and heat transfer rates as well as on the reaction equilibrium and kinetics, represented by the equations that describe mass and energy balances, mass transfer rates for each component, and reaction rate-related equations for each reaction occurring in the reactor.^20^

Ghemei and Shahhosseini^21^ proposed a mathematical model of imbalance based on a model of a dynamic film for the reactive absorption of hydrogen sulfide in diglicolamine solution, considering simultaneous transfers of heat and mass and reaction rates. The results showed that, owing to the increased pressure in the column, the temperature of the liquid phase and the concentration of amine as well as the liquid flow rate could increase the absorption capacity of the system.

A model based on the two-film theory was developed by Bontozoglou and Karabelas^13^ to predict the simultaneous absorption of H_2_S and CO_2_ in some aqueous alkaline solutions. Since acid gas absorption is an exothermic process, elevated temperatures (e.g., 200 °C) reduce the solubility of CO_2_ and H_2_S, thereby impairing absorption efficiency. As a result, gas–liquid mass transfer and selectivity are more favorable at lower temperatures and are primarily governed by reaction kinetics, solvent properties, and process conditions. This thermodynamic behavior has been consistently observed in equilibrium and kinetic modeling studies (Luiz de Medeiros et al.; Maile et al.),^22,23^ and confirmed experimentally for sodium hydroxide systems (Aliev et al.).^24^ Furthermore, recent advances in solvent engineering, including lithium-enhanced MEA formulations and amino-acid-functionalized sorbents, have highlighted strategies to maintain performance under temperature stress (Ismail et al.; Mohammed Hatta et al.).^25,26^ Mohammad et al.^27^ developed a mathematical model by applying computational fluid dynamics (CFD) techniques for the simultaneous chemical absorption of CO_2_ and H_2_S in monoethanolamine (MEA). It was found that thermal effects were significant for high gas velocities or small liquid phase velocities and played an important role in the model predictions.

Another mathematical model in this context was proposed by Bashipour et al.^28^ to represent the reactive absorption of H_2_S in NaOH, sodium hypochlorite or H_2_O_2_. The model predicts the effects of operating variables such as the concentration of H_2_S entering the equipment, gas flow rate, initial concentration of NaOH, concentrations of oxidizing agents in washing solutions and liquid-to-gas ratio on the H_2_S removal efficiency.

The modeling of H_2_S absorption in gas treatment has evolved from simplified equilibrium-based formulations to detailed kinetic and transport-based approaches. Hashemi et al.^16^ demonstrated the importance of multispecies diffusion and heat effects using CFD in MEA systems. Bontozoglou and Karabelas^13^ emphasized kinetic selectivity for H_2_S in hydroxide media. However, these works remain anchored in steady-state assumptions and do not readily enable dynamic simulation or control applications.

More recent contributions, such as the oxidative kinetic modeling by Ali et al.^17^ and radical pathway studies by Lai et al.,^18^ reflect the increasing complexity of reactive systems. In particular, Cao et al.^19^ introduced new methods to evaluate oxidation in sulfhydryl-containing systems, showing the importance of mass transfer limitations. Despite these advances, there is still a lack of accessible, real-time predictive models for alkaline scrubbing systems suitable for remote gas treatment infrastructure. This work fills that gap by offering a validated, time-resolved simulation tool based on measurable and controllable parameters, most notably solution pH.

## 2 Methodology

### 2.1 Experimental Data

The experimental apparatus was designed to operate in a semi batch system consisting of a bubble column containing a certain volume of an aqueous sodium hydroxide solution in which natural gas flow atomized by a sintered glass diffuser was bubbled, as shown in Figure 1. The experimental setup consists of a vertical absorption column constructed from rigid PVC, with an internal diameter of 31/2 in (approximately 8.9 cm) and a total height of 100 cm. A sintered glass diffuser (pore size: 45 μm) is installed at the base to ensure uniform gas dispersion. The column has threaded connections at both ends and is connected to a gas supply via needle and ball valves made from brass/stainless steel. A Bourdon-type manometer (0–5 kgf/cm^2^) measures system pressure. The gas phase, composed of synthetic air with H_2_S concentrations between 855.7 and 1491.0 ppm, is delivered using a pressurized cylinder and a White Martins rotameter (capacity up to 14 L/min). The liquid phase is a 1.0 mol/L NaOH solution, recirculated through the packed section at 1.0 L/min using a peristaltic pump. The system is operated isothermally at 25  ±  1 °C. Measured parameters include time-resolved pH (accuracy ± 0.01), H_2_S concentration in the gas outlet using calibrated detection tubes, and sulfide accumulation in the liquid phase. Although the gas stream contains CO_2_ to simulate natural gas composition, no direct measurements or simulations were conducted for CO_2_ absorption, as the study focuses exclusively on H_2_S kinetics. The operating conditions are summarized in Table 1.

**Figure 1:**
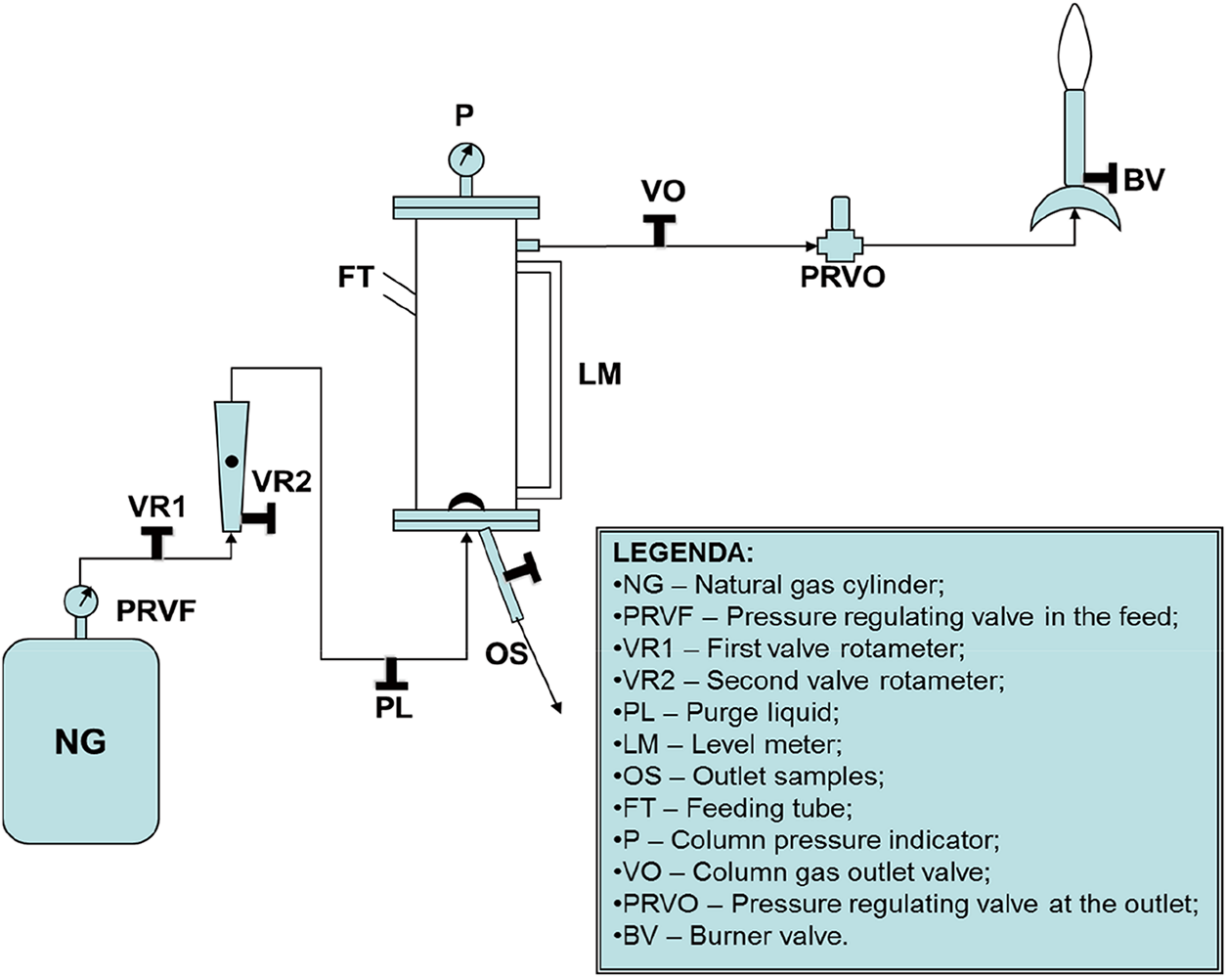
Sketch of the experimental apparatus.

**Table 1:** Experimental Data Set

test	*Q* (m^3^/s ) × 10^5^	*V* (m^3^) × 10^3^	*C*_H_2_S_ (mol/m^3^) × 10^2^	C_NaOH_ (mol/m^3^)
1	3.33	2.0	3.50	1000.0
2	3.33	2.0	6.09	1000.0
3	6.67	2.0	6.09	1000.0
4	5.00	2.0	6.09	1000.0

In this study, the model assumes isothermal operation at 25 ±  1 °C. Thermal effects, including temperature-dependent solubility and reaction kinetics, are not explicitly considered but are acknowledged as critical for future model development.

### 2.2 Model Development

All simulations were conducted using EMSO (Environment for Modeling, Simulation and Optimization), an open-source, equation-oriented simulation platform developed by the ALSOC Project at UFRGS (Brazil). EMSO supports the formulation and numerical solution of systems of differential-algebraic equations (DAEs) using symbolic differentiation, automatic index reduction, and unit consistency enforcement (Soares and Secchi).^29^ It allows dynamic and steady-state modeling through modular, object-oriented structures. EMSO has been successfully applied in various domains, such as compressor dynamics in gas processing units (Cunha et al.),^30^ gasification of municipal solid waste (Oliveira et al.),^31^ catalytic reaction modeling (Silva et al.),^32^ and optimization of large-scale process systems (Henrique et al.).^33^ These applications demonstrate EMSO’s maturity as a tool for predictive, transient simulations essential to natural gas treatment and acid gas removal systems (Qayyum et al.).^34^

The EMSO simulator uses an object-oriented programming language that allows users to develop complex models by combining simpler models.^35^ The computational domain and systematics used to solve the equation system are shown in Figure 2.

**Figure 2:**
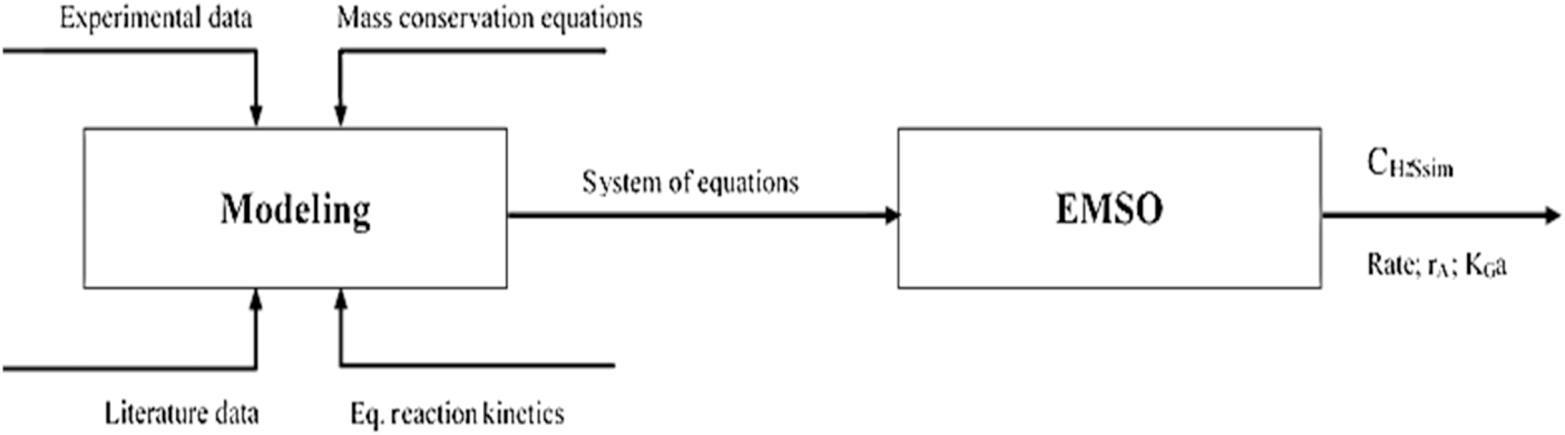
Flowchart of theoretical and computational planning.

Kinetic models for mass transfer and the reactive absorption process were implemented in EMSO by considering the stoichiometry of multiple reactions. Thermodynamic properties of the components, obtained from the literature and experimental data, were supplied to the EMSO solver to enable model parameter estimation. The pseudo reactor configuration and the simplifying assumptions are shown in Figure 3. Figure 3 illustrates the absorption system, where raw natural gas enters from the bottom, passes through a packed column under dynamic conditions, and exits as treated natural gas. The surrounding blocks identify the system’s kinetic assumptions, operational parameters, and the physical–chemical data required for model formulation.

**Figure 3:**
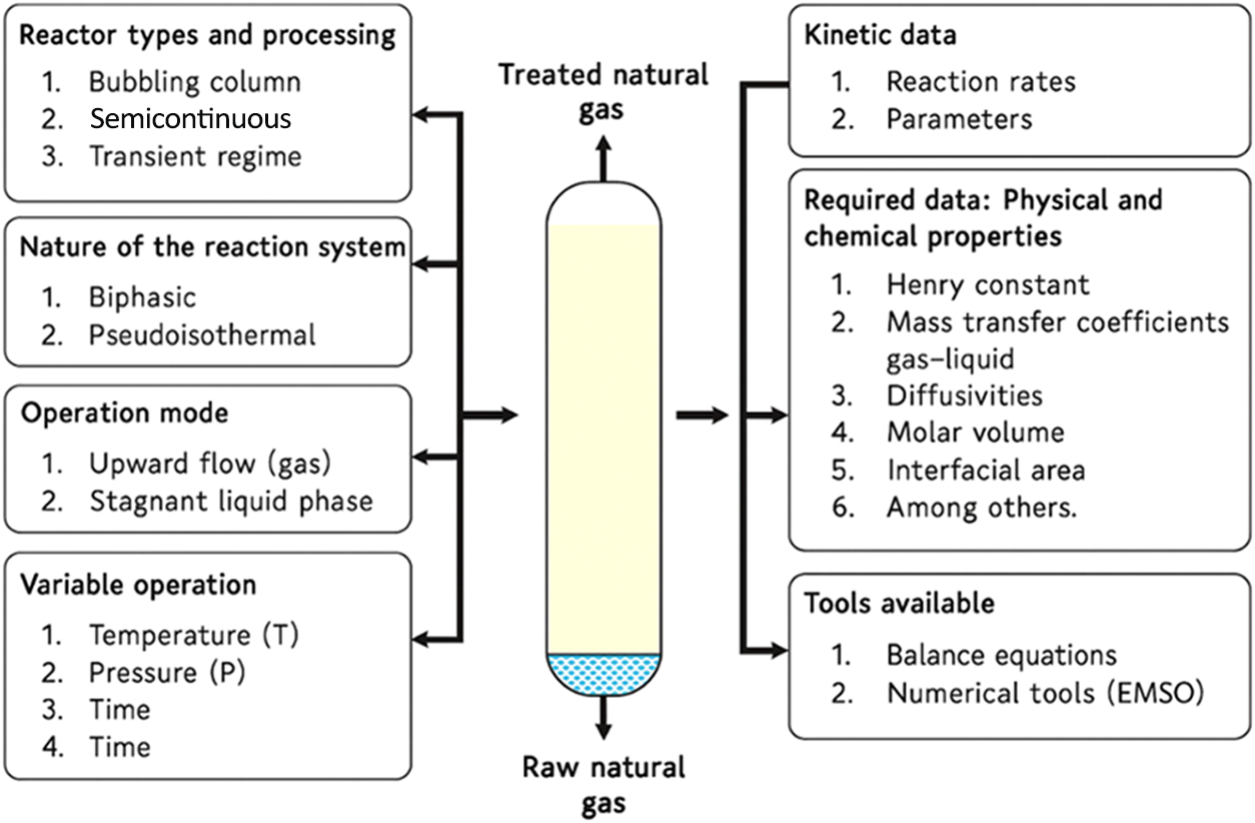
Operating assumptions for the modeling process.

Thermal effects were not considered due to the absence of experimental data for validation, but they are implicitly accounted for through the values of the parameters and experimental measurements used. The absorber was operated in semi batch mode with respect to the liquid phase and in continuous mode with respect to the gas phase. Other relevant considerations include the use of cylindrical coordinate systems (*z*, *r*, and θ), constant pressure, and uniform spherical bubbles of constant diameter.

#### 2.2.1 Reaction Mechanism

The absorption of hydrogen sulfide (H_2_S) in aqueous sodium hydroxide (NaOH) occurs through a series of acid–base neutralization reactions. Under strongly alkaline conditions, these reactions are fast and can be approximated as irreversible

1 $${H_{2}S}_{\left(\mathrm{aq}\right)}+{\mathrm{OH}}^{-}\to {\mathrm{HS}}^{-}+H_{2}O$$

2 $${\mathrm{HS}}^{-}+{\mathrm{OH}}^{-}\to S^{2-}+H_{2}O$$

Similarly, CO_2_ absorption follows the neutralization pathway

3 $${{\mathrm{CO}}_{2}}_{\left(\mathrm{aq}\right)}+{\mathrm{OH}}^{-}\to {\mathrm{HCO}}_{3}^{-}$$

4 $${\mathrm{HCO}}_{3}^{-}+{\mathrm{OH}}^{-}\to {\mathrm{CO}}_{3}^{2-}+H_{2}O$$

Due to the high pH maintained during absorption (pH > 11), these reactions are assumed to be thermodynamically driven to completion, justifying a pseudoirreversible treatment in the kinetic model. This simplification is supported by literature studies such as Luiz de Medeiros et al.,^22^ Aliev et al.,^24^ and Coquelet et al.^36^

#### 2.2.2 Reactor Model and Flow Conditions

The reactor is modeled as a vertical bubbling column, where gas flows upward through a stagnant liquid phase. The gas enters as a dilute H_2_S-air mixture and is distributed via a porous diffuser. The liquid phase (NaOH solution) is well mixed due to recirculation, and the absorption process is considered pseudo homogeneous in the bulk phase. The system is assumed isothermal (25  ±  1 °C), and pressure effects are neglected due to the low operating pressure (∼1.0  ±  0.1 kgf/cm^2^).

#### 2.2.3 Mass and Charge Balances

Based on the reactor configuration and assumptions, the following transient mass balances are formulated for the liquid phase

5 $$\frac{dC_{H_{2}S}}{dt}=-k_{1}C_{H_{2}S}C_{{OH}^{-}}$$

6 $$\frac{dC_{{\mathrm{HS}}^{-}}}{dt}=k_{1}C_{H_{2}S}C_{{\mathrm{OH}}^{-}}-k_{2}C_{\mathrm{HS}}C_{{\mathrm{OH}}^{-}}$$

7 $$\frac{dC_{S^{2-}}}{dt}=k_{2}C_{\mathrm{HS}}C_{{\mathrm{OH}}^{-}}$$

Similarly, the balance for OH^–^ is given by

8 $$\frac{dC_{{\mathrm{OH}}^{-}}}{dt}={-k}_{1}C_{H_{2}S}C_{{\mathrm{OH}}^{-}}-k_{2}C_{\mathrm{HS}}C_{{\mathrm{OH}}^{-}}+R_{\mathrm{addition}},\left(\mathrm{if}\;\mathrm{replenished}\right)$$

Charge balance and pH evolution are tracked by linking OH^–^ depletion to H^+^ formation using the relation

9 $$\mathrm{pH}=14+\log_{10}\left([C_{{\mathrm{OH}}^{-}}]\right)$$

#### 2.2.4 Initial and Boundary Conditions

Initial concentrations are based on known input concentrations at *t* = 0

10 $$C_{H_{2}S}=C_{H_{2}S,\mathrm{in}}$$

11 $$C_{{\mathrm{OH}}^{-}}=1000.0\;\left(\mathrm{mol}/m^{3}\right)$$

12 $$C_{{\mathrm{HS}}^{-}}=0,C_{S^{2-}}=0$$

Boundary conditions for gas inlet concentration, flow rate, and pH evolution follow the operating setup described in Section 2.1.

#### 2.2.5 Integrated Kinetic–Transport Equations

To couple the kinetic expressions with mass transport, the following equations govern the dynamic behavior of H_2_S in the gas and liquid phases:

The mass balances of H_2_S in the gas and liquid phases are expressed by eq 13 and eq 14, respectively

13 $$\frac{dC_{H_{2}S}}{dt}=Q_{\mathrm{vaz}}.\left(C_{H_{2}S}^{0}-C_{H_{2}S}\right)-\mathrm{rate}-r_{A}$$

14 $$\frac{dC_{\mathrm{sol}}}{dt}=r_{A}+\mathrm{rate}$$

where the mass transfer rate is given by eq 15

15 $$\mathrm{rate}=K_{G}a.\left(C_{H_{2}S}-C_{H_{2}S}^{*}\right)$$

where *C*_H_2_S_ is the concentration at an arbitrary instant t, *C*_H_2_S_^*^ is the concentration at equilibrium, *C*_H_2_S_^0^ is the concentration entering the column, *C*_sol_ is the bulk liquid phase concentration, *K*_G_ *a* is the global volumetric mass transfer coefficient in the gas phase and *k* is the reaction rate constant.

According to Luyben,^37^ with respect to the Guldberg-Waage law, the overall reaction rate of elementary reactions varies simultaneously with temperature, and the product of the reagent concentrations increases to their respective stoichiometric coefficients (eq 16).

16 $$r_{A}=-k.C_{\mathrm{sol}}^{a}.C_{H_{2}S}^{b}$$

At equilibrium, concentrations can be expressed in terms of Henry’s law, according to eq 17.

17 $$C_{H_{2}S}^{*}=H_{A}.C_{\mathrm{sol}}$$

These relations allow simultaneous computation of chemical conversion, absorption rate, and system dynamics over time. The reaction order (a, b) and constants (*k*, *H*_*A*_) are evaluated under the alkaline conditions described in Section 2.1. Although the primary objective of this study is to simulate the dynamic absorption of acid gases in alkaline media, the kinetic formulation remains fundamentally constrained by underlying thermodynamics. In particular, the acid–base reactions between CO_2_, H_2_S, and hydroxide ions are treated as irreversible under the high-pH conditions considered in this work. This modeling assumption is widely supported in the literature, as strong alkaline environments drive the formation of stable ionic products such as HCO_3_^–^, CO_3_^2–^, HS^–^, and S^2–^, shifting equilibrium far toward completion.

Coquelet et al.^36^ demonstrated that both CO_2_ and H_2_S show effectively irreversible absorption behavior in highly basic aqueous media due to thermodynamic and kinetic favorability. Luiz de Medeiros et al.^22^ provided a rigorous equilibrium-based parametrization for alkanolamine systems, confirming that reverse reactions are negligible at elevated pH. Experimental observations by Aliev et al.^24^ further support this assumption, showing complete neutralization of H_2_S with NaOH under alkaline gas scrubbing conditions.

Accordingly, our reaction kinetics are framed within these thermodynamic constraints, assuming a pseudoirreversible progression to product formation under conditions of excess OH^–^ availability and ambient temperature. This treatment is appropriate for dynamic modeling in systems where equilibrium is effectively unattainable during the short residence times or rapid absorption events being studied.

While the proton transfer reaction between H_2_S and OH^–^ is well-known to be extremely rapid and is often treated as equilibrium-controlled, we adopt a first-order kinetic approximation to capture transient effects during gas–liquid contact. This allows the model to reflect time-dependent changes in pH and reactant concentrations, particularly under dynamic flow or variable loading conditions. This approach serves as a computationally efficient compromise between mechanistic fidelity and predictive capability in real-time process modeling.

## 3 Results and Discussion

### 3.1 Model Validation

The calibration of the model for the reactive absorption of H_2_S in aqueous NaOH solution discussed above was carried out by using experimental data to estimate the kinetic reaction rate constant and global volumetric mass transfer coefficient on the gas phase side, as shown in Table 2. These parameters enable the model to predict the behavior of the system under diverse operating conditions.

**Table 2:** Model Parameters Estimated in the Calibration Step

test	*k* (mol·*s* ^–1^·m^–3^)	*K*_G_ *a* (s^–1^)	*R* ^2^
1	8.59 × 10^–03^	6.15 × 10^–04^	0.97879
2	2.24 × 10^–04^	5.12 × 10^–04^	0.82507
3	7.43 × 10^–06^	6.39 × 10^–04^	0.99196
4	6.12 × 10^–02^	8.58 × 10^–04^	0.90366

Figures 4 and 5 show the validation results for the reactive absorption of H_2_S, confirming that the model is capable of accurately predicting process behavior.

**Figure 4:**
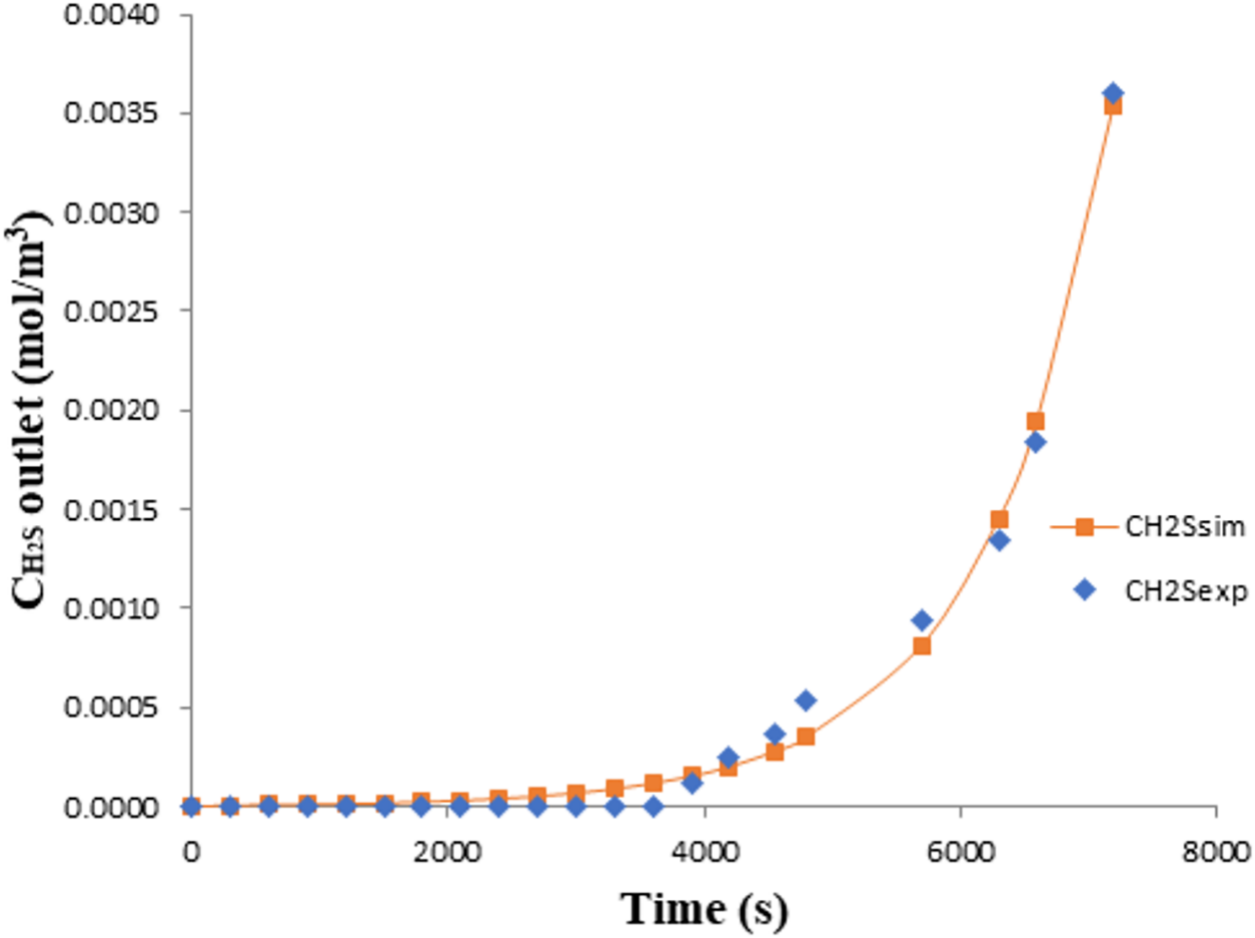
Model validation for the reactive absorption of H_2_S in NaOH for experimental run number 3; *C*_H_2_S_ = 6.09 × 10^–2^ mol·m^–3^; *Q* = 3.33 × 10^–5^ m^3^·s^–1^ *C*_NaOH_ = 1000 mol·m^–3^; *V* = 2.0 × 10^–3^ m^3^.

**Figure 5:**
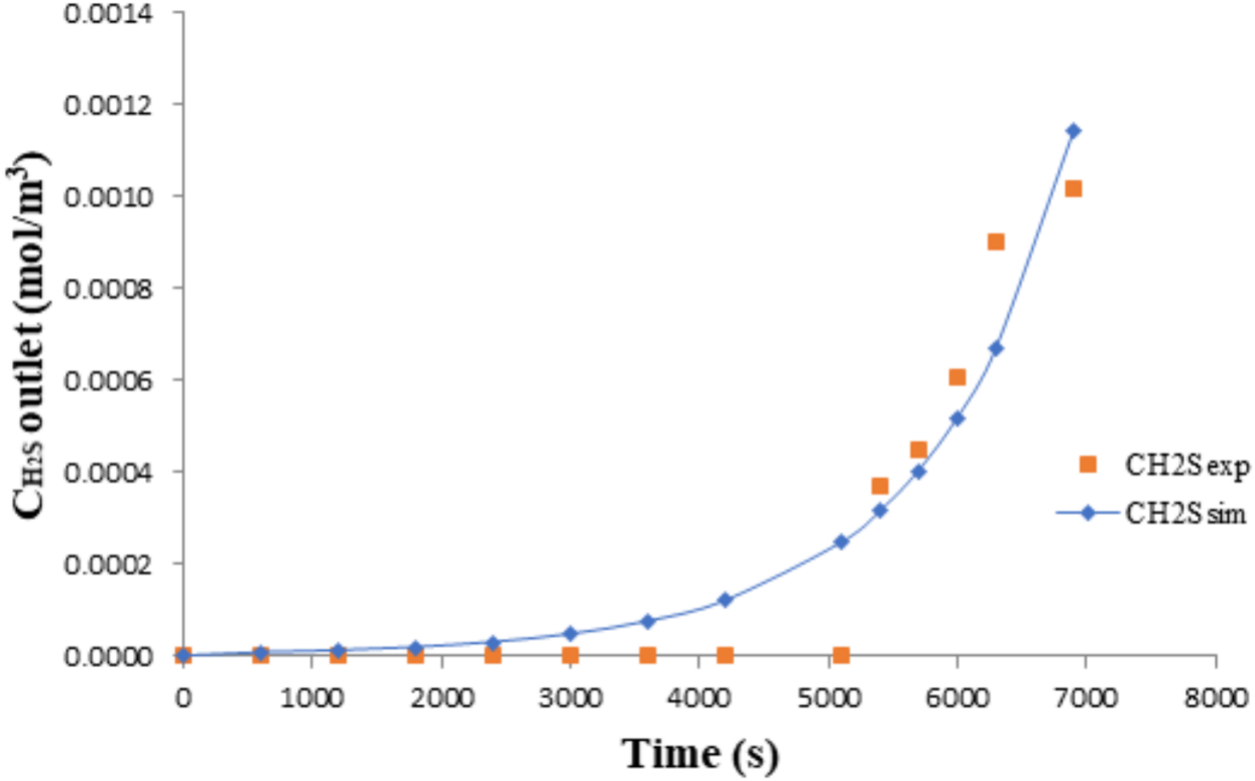
Model validation for the reactive absorption of H_2_S in NaOH for experimental test data 4. *C*_H_2_S_ = 6.09 × 10^–2^ mol·m^–3^; *Q* = 5.00 × 10^–5^ m^3^·s^–1^; *C*_NaOH_ = 1000 mol·m^–3^; *V* = 2.0 × 10^–3^ m^3^.

### 3.2 Evaluation of Reactive Absorption of H_2_S BY NaOH

Figures 4 and 5 present the pH decay profile and sulfide concentration profile, respectively, for the absorption of H_2_S in NaOH solution. In both cases, the continuous lines represent model predictions, while the data points correspond to experimental results for runs 3 and 4, as previously defined. The observed system behavior indicates that absorption is not governed by physical solubility alone. Instead, a dominant chemical reaction occurs between H_2_S and the alkaline absorbent, which substantially enhances the mass transfer rate. Nearly all H_2_S transferred into the liquid phase reacts rapidly with NaOH, as seen in the initial steep portion of the curves. As hydroxide ions are consumed, the mass transfer driving force decreases, and the curves begin to deviate from their initial trajectories, signaling the onset of mass transfer limitation. Near equilibrium, as the chemical absorption capacity is exhausted, physical absorption becomes dominant and the net H_2_S uptake rate approaches zero.

In other words, the rapid initial drop in pH and high uptake rate of H_2_S, as shown in Figures 4 and 5, reflect the dominance of chemical absorption under strongly alkaline conditions. Nearly all H_2_S transferred to the liquid phase reacts promptly with hydroxide ions, justifying the use of a pseudoirreversible kinetic model. As hydroxide is progressively consumed, the system transitions toward equilibrium and the absorption rate decreases, consistent with a declining mass transfer driving force. These trends validate the model structure and confirm that reaction kinetics govern the system’s transient behavior during the absorption process.

Vogel^38^ indicates that some possible reactions involve the species H_2_S and CO_2_, which is very useful in the present analysis once the raw natural gas contains these two substances, as follows:

(i) CO_2_ absorption reaction:
18 $${\mathrm{CO}}_{2}+2OH^{-}⇌CO_{3}^{2-}+H_{2}O$$
(ii) H_2_S absorption reaction:
19 $$H_{2}S+2OH^{-}⇌S^{2-}+2H_{2}O$$
(iii) Hydrolysis of CO_3_^2–^:
20 $$CO_{3}^{2-}+H_{2}O⇌\mathrm{HC}O_{3}^{-}+OH^{-}$$
(iv) Hydrolysis of *S* ^2–^:
21 $$S^{2-}+H_{2}O⇌HS^{-}+OH^{-}$$
(v) Reaction between CO_3_^2–^ and H_2_CO_3_:
22 $$CO_{3}^{2-}+H_{2}CO_{3}⇌2\mathrm{HC}O_{3}^{-}$$
(vi) Reaction between S^2–^ and H_2_S:
23 $$S^{2-}+H_{2}S⇌2HS^{-}$$
(vii) Reaction between H_2_S and CO_3_^2–^:
24 $$H_{2}S+2CO_{3}^{2-}⇌2\mathrm{HC}O_{3}^{-}+S^{2-}$$
(viii) Reaction between H_2_S and HCO_3_^–^:
25 $$H_{2}S+2\mathrm{HC}O_{3}^{-}⇌2H_{2}CO_{3}+S^{2-}$$

After careful analysis of the values of the equilibrium constants for the reactions above, the following reactions are considered the most likely:

26 $$\begin{array}{ll} {\mathrm{CO}}_{2}+2OH^{-}⇌CO_{3}^{2-}+H_{2}O & K=O\left(10^{11}\right) \end{array}$$

27 $$\begin{array}{ll} S^{2-}+H_{2}S⇌2HS^{-} & K=O\left(10^{8}\right) \end{array}$$

28 $$\begin{array}{ll} H_{2}S+2OH^{-}⇌S^{2-}+2H_{2}O & K=O\left(10^{6}\right) \end{array}$$

29 $$\begin{array}{ll} CO_{3}^{2-}+H_{2}CO_{3}⇌2\mathrm{HC}O_{3}^{-} & K=O\left(10^{4}\right) \end{array}$$

30 $$\begin{array}{ll} H_{2}S+2CO_{3}^{2-}⇌2\mathrm{HC}O_{3}^{-}+S^{2-} & K=O\left(10^{-2}\right) \end{array}$$

31 $$\begin{array}{ll} H_{2}S+2\mathrm{HC}O_{3}^{-}⇌2H_{2}CO_{3}+S^{2-} & K=O\left(10^{-35}\right) \end{array}$$

Therefore, reactions 26 to 31 must predominate in the system, inferring that the exhausted absorber solution must contain predominantly bicarbonate and disulfide ions, ensuring that bicarbonate species are probably the only H_2_S consumer near equilibrium. Although the raw natural gas used in the experiments contains CO_2_, its action inherent to the consumption of NaOH is transitory because the reaction of hydroxyl ions with carbon dioxide generates carbonate and bicarbonate ions. These secondary species are able to react with H_2_S, resulting in an overwhelming reaction between H_2_S and NaOH.

### 3.3 Effect of the Natural Gas Flowrate on the H_2_S Absorption Efficiency

One of the main goals of any mathematical model is to predict the system behavior in situations diverse from those in laboratories. To determine the influence of the natural gas flow on the reactive absorption of H_2_S while keeping the concentration of the absorbing solution constant, three situations were run, resulting in the results shown in Figure 6.

**Figure 6:**
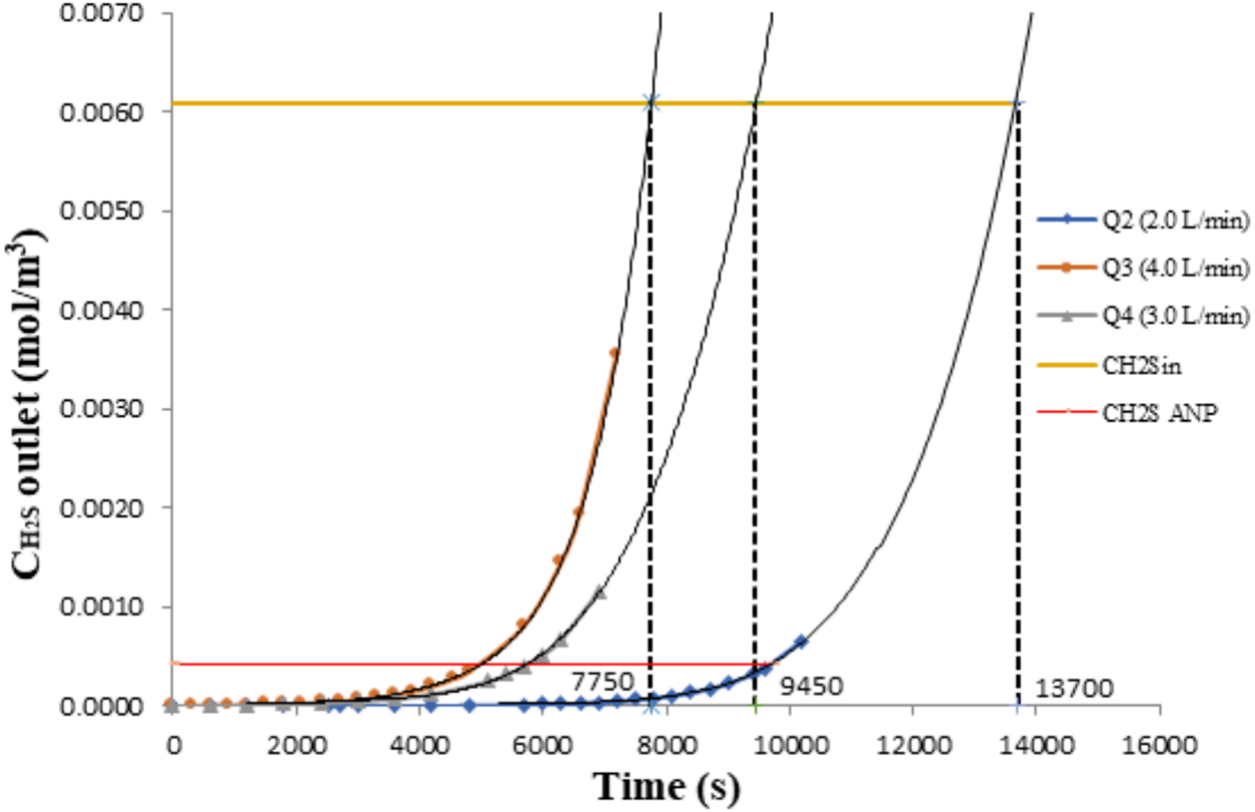
Influence of the natural gas feed flow on the saturation time of the absorbing solution. *Q*_vaz2_ = 3.33 × 10^–5^ m^3^·s^–1^; *Q*_vaz3_ = 6.67 × 10^–5^ m^3^·s^–1^; and *Q*_vaz4_ = 5.00 × 10^–5^ m^3^·s^–1^.

A trendline of the fifth-degree polynomial type was adopted to predict but not explain the process to fit the experimental data, allowing extrapolation to estimate the instant when saturation occurs, represented by the line corresponding to C_H2S_ = 6.09.10^–3^ mol·m^–3^, which is the concentration of H_2_S in the raw gas. From this instant on, the solute is no longer absorbed.

In practice, process operation must be stopped before saturation because the concentration of H_2_S in the exit gas is the limiting factor established by legislation. In Brazil, the National Agency of Petroleum and Gas (ANP) determines threshold concentrations for all contaminants in processed natural gas.^39^

Simulations involving absorbing solution saturation time were also run to predict NaOH concentration behavior during operation, as shown in Figure 7. A comparison of the results depicted in Figures 6 and 7 reveals that they are well correlated, indicating the consistency of the model.

**Figure 7:**
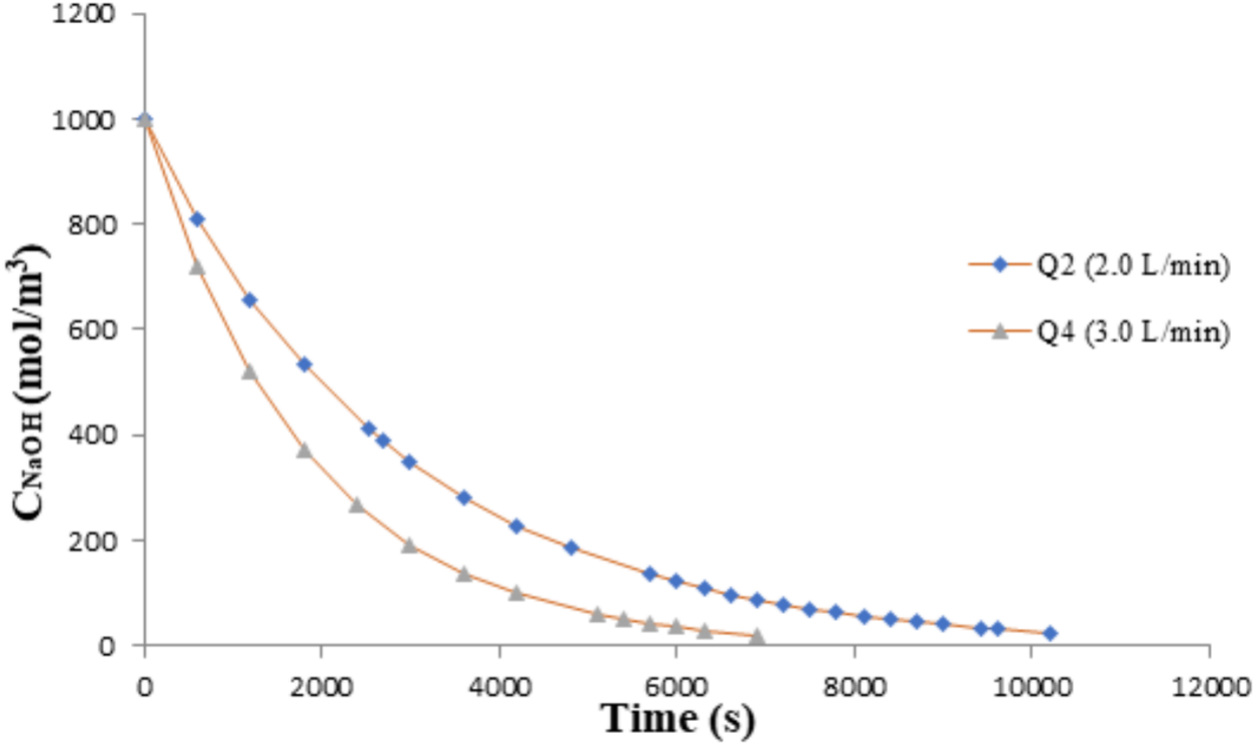
Influence of the natural gas feed flow rate on the saturation time of the absorbing solution. *Q*_vaz2_ = 3.33 × 10^–5^ m^3^·s^–1^; *Q*_vaz3_ = 6.67 × 10^–5^ m^3^·s^–1^.

### 3.4 Evaluation of the Reaction Rate in the Process

The fundamental principles governing the reaction rate of H_2_S in alkaline scrubbing systems have long been established. In this section, we aim not to introduce new chemical mechanisms but to demonstrate how the proposed kinetic model quantitatively reproduces classical behavior predicted by two-film theory and verified through decades of experimental work. In the context of gas–liquid absorption, a high chemical affinity between solute and solvent results in a rapid consumption of the solute in the liquid phase. This generates a steep concentration gradient near the interface, which in turn sustains a high mass transfer rate from the gas phase. For systems such as H_2_S absorption in NaOH, the reaction is known to be fast and effectively irreversible under strongly alkaline conditions. As a result, the rate of mass transfer becomes closely coupled to the chemical reaction rate.

Figure 8 illustrates this relationship by comparing the predicted mass transfer rate and reaction rate for H_2_S under typical operating conditions. The near-overlap of the curves indicates that the absorbed H_2_S is immediately consumed in the bulk liquid, maintaining a low concentration near the interface. This observation is fully consistent with the classical two-film theory, which predicts that when chemical reaction is significantly faster than diffusion, the reaction zone shifts to the liquid boundary layer. These results confirm that the model correctly captures the interplay between transport and reaction phenomena in alkaline scrubbing systems. This validation supports its use for real-time simulation, design, and control of field-scale treatment operations.^40^

**Figure 8:**
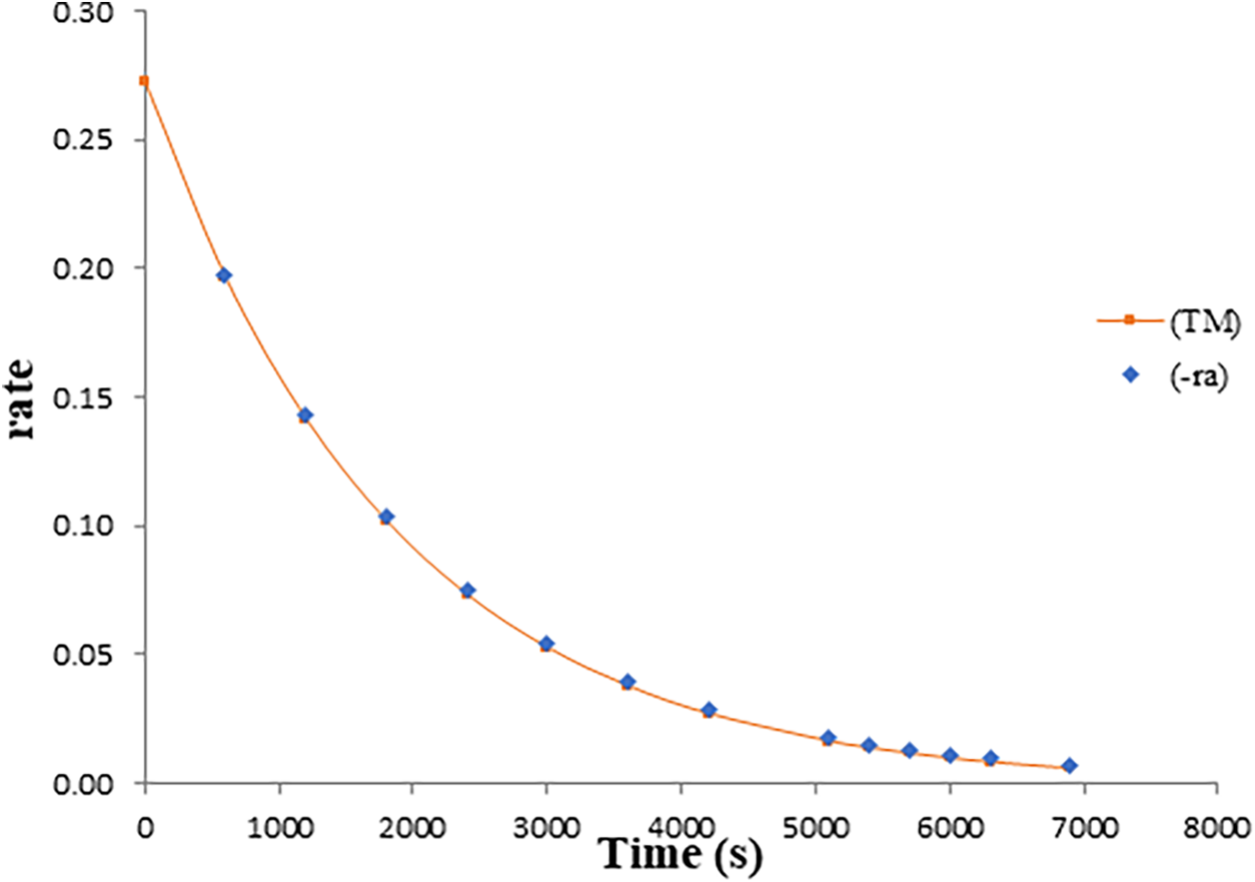
Comparison between the mass transfer rates and reactions during the reactive absorption of H_2_S in NaOH solution; *C*_H2S_ = 6.09 × 10^–2^ mol·m^–3^; *Q* = 5.00 × 10^–5^ m^3^·s^–1^; *C*_NaOH_ = 1000 mol·m^–3^; *V* = 2.0 × 10^–3^ m^3^.

### 3.5 Behavior of pH in the Reactive Absorption

Figure 9 shows the actual values of pH and exit H_2_S concentrations obtained experimentally, as discussed above, in addition to the H_2_S concentration values predicted by the model throughout the operating time. These combined results for the pH of the absorber solution and H_2_S output concentrations are consistent with the experimental results. The actual pH for H_2_S detection in the exit gas was approximately 10. According to ANP,^39^ the specification for the maximum concentration of H_2_S in natural gas must not exceed 10.0 ppm (4.09 × 10^–4^ mol·m^–3^), which, in Figure 9, is registered for a pH value and saturation time of approximately 9.6 and 85 min, respectively. If the operation is interrupted at pH 10, we can ensure the attainment of the ANP’s specification. The more important issue regarding the results shown in Figure 9 is the possibility of using pH as the only control variable, with the additional advantage of being a very easy-to-measure parameter.

**Figure 9:**
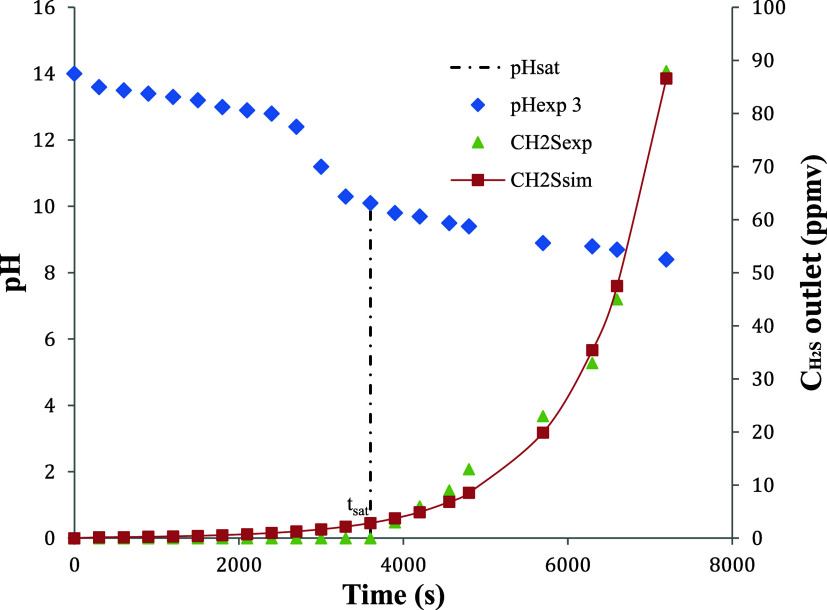
Behavior of the pH in the saturation curve of the absorbing solution to experimental test data 3; *C*_H2S_ = 6.09 × 10^–2^ mol·m^–3^; *Q* = 3.33 × 10^–5^ m^3^·s^–1^; *C*_NaOH_ = 1000 mol·m^–3^; *V* = 2.0 × 10^–3^ m^3^.

According to Figure 9, considering that the raw gas contains CO_2_ in addition to H_2_S as sour gases, at pH values between 10 and 14, reactions (i), (ii) and (vii) are predominant. Moreover, for pH values below 10, the reaction (viii) must predominate, suggesting that the presence of CO_2_ does not interfere substantially with the overall absorption of H_2_S, since the reaction between CO_2_ and NaOH produces alkaline species that have high chemical affinity with H_2_S.

## 4 Conclusions

A transient mathematical model was developed to predict the absorption dynamics of hydrogen sulfide (H_2_S) in an aqueous sodium hydroxide (NaOH) solution. The model captures the effects of key variables such as sour gas concentration, gas flow rate, and NaOH concentration, and it simulates the evolution of H_2_S and NaOH concentrations, as well as pH over time.

The following conclusions can be drawn from the modeling and experimental results:

- The proposed model demonstrated strong predictive performance under both calibrated and extrapolated operating conditions, matching well with experimental data.
- Key parameters-namely the kinetic reaction rate constant and the global volumetric mass transfer coefficient in the gas phase-were successfully estimated and found to be consistent with literature values.
- The effects of gas flow rate, reaction kinetics, and pH on H_2_S removal efficiency were systematically analyzed.
- While CO_2_ was present in the synthetic gas mixture to reflect natural gas composition, no direct measurement or simulation of CO_2_ absorption was conducted. Nevertheless, the model results qualitatively align with the expected selectivity of NaOH for H_2_S under strongly alkaline conditions.
- The model confirms that pH and outlet H_2_S concentration are both effective variables for monitoring and controlling the absorption process.
- The predicted saturation times of the absorbent solution closely matched experimental observations, further validating the model’s transient accuracy.

These results provide a strong foundation for guiding future work in the modeling, simulation, and optimization of reactive absorption systems for acid gas removal. The proposed model integrates key chemical kinetics with operational parameters in a time-resolved framework, enabling dynamic analysis of process performance. This approach can support the development of advanced predictive tools for alkaline scrubbing, including the evaluation of temperature effects, multicomponent gas mixtures, and system-scale optimization for industrial or field-scale applications.

## Data Availability

The data will be made available upon request.
